# The Role of External Fixation in the Treatment of Distal Radius Fractures

**DOI:** 10.7759/cureus.64346

**Published:** 2024-07-11

**Authors:** Robert Kamil, Elise McKenna, Paul Romeo, Orett Burke, Anna Zakusylo, Aman Andemichael, Nicole Badalyan, Thomas Stamos, Ajul Shah, Brian M Katt

**Affiliations:** 1 Orthopedic Surgery, Rutgers Robert Wood Johnson Medical School, New Brunswick, USA; 2 Orthopedic Surgery, Rutgers New Jersey Medical School, Newark, USA; 3 Plastic Surgery, Center for Hand and Upper Extremity Surgery, Shrewsbury, USA

**Keywords:** polytrauma, dorsal spanning plates, internal fixation, external fixation, fracture, distal radius

## Abstract

There are numerous internal fixation (IF) options available for distal radius fractures (DRFs). The choice of fixation method depends on factors such as fracture morphology, soft tissue integrity, the patient's clinical status, and the surgeon's training. While volar plate fixation has become the primary approach for addressing these fractures, alternative IF methods like K-wire fixation, fragment-specific fixation, and dorsal bridge plating continue to be effective. Despite the versatility of IF, there are certain clinical situations where prompt and conclusive management through open reduction and internal fixation (ORIF) is not suitable. These instances include the treatment of polytraumatized patients, individuals with compromised soft tissues, or those medically unstable to tolerate lengthy anesthesia. In such cases, proficiency in closed reduction and external fixation (EF) proves invaluable. Being able to identify these clinical scenarios and comprehend the efficacy and safety of EF in addressing DRFs is valuable for any surgeon handling such injuries.

## Introduction and background

Distal radius fractures (DRFs) are the most common upper extremity fractures in the United States [1]. Optimal treatment of radiographically confirmed DRFs is often guided by numerous factors, including patient demographics (age, gender, activity level), as well as radiographic appearance (fracture displacement, angulation, comminution, and intra-articular involvement). Closed, displaced DRFs are appropriately managed with definitive closed reduction and immobilization if post-reduction imaging parameters show no more than 3 mm of radial shortening, less than 10 degrees of dorsal angulation, and less than 2 mm of intra-articular displacement [2].

While a pragmatic approach often involves attempting closed reduction first, it is recognized that certain fractures, due to their complexity or patient-specific factors, may necessitate direct surgical intervention without prior closed reduction attempts. Fractures that do not meet the required parameters after closed reduction are generally indicated for surgery [3]. Surgical treatment has steadily increased from 8% in 1997 to 24% in 2018 with the rate in the elderly as high as in the non-elderly [4]. In 1996, the treatment distribution for those treated operatively was 59% external fixation (EF), 20% plate fixation, and 18% K-wire fixation. While in 2018, 96% of patients operated upon were treated with plate fixation [4,5]. As the incidence of these complex fractures has increased, strong evidence favoring the use of volar locking plates (VLP) in immediate open reduction internal fixation (ORIF) has also increased [5]. The current orthopedic literature highlights that VLP ORIF yields the best functional outcomes with lower rates of complications, infections, and cost relative to other forms of plating and fixation techniques [6-9].

However, high-energy comminuted fractures with or without meta diaphyseal extension and operating on unstable patients remain major treatment challenges. Dorsal distraction plating, commonly referred to as bridge plating, was first described in a case report by Burke and Singer in 1998 and has emerged as an alternative internal fixation (IF) technique [10]. When combined with reduction of the articular surface and bone grafting, it has proven to be a successful alternative to VLP in providing acceptable reduction and functional range of motion for comminuted intra-articular fractures [11]. While the evidence in support of bridge plating continues to rise, there remain certain drawbacks regarding cost and the need for a second procedure to remove the plate.

EF is an alternative surgical technique that has become less common in treating DRFs over the past several years [4,5]. This may largely be due to improved IF methods and attempts to avoid EF complications that include malunion, infections, hardware failure, and complex regional pain syndrome [2,6]. Yet, EF remains a preferred method of management by some providers who claim that it provides the best fixation for intra-articular DRFs. In the paper by Ma et al., the plating group had a greater occurrence of wound infection, tendonitis, and additional surgery compared with the EF group [12].

In the evaluation of treatment modalities for DRFs, there currently exists much debate regarding the implementation of external fixators. Few studies have elaborated on their necessity, and several questions still need to be addressed as practitioners seek consensus on treatment protocols for these fractures. The goal of this narrative review is to describe the clinical efficacy and safety of EF. Providing surgeons with this information backed by the literature will help them decide when EF may be a suitable alternative to IF techniques.

## Review

Commonly agreed-upon external fixation indications

Temporary Fixation

The orthopedic literature reflects several points of distal radius consensus concerning EF. Immediate closure is not always possible, especially under circumstances like injuries that occur in war zones and those resulting from natural disasters. Such conditions spawned the concept of damage control orthopedics (DCO), which is a series of protocols intended for polytrauma patients with extensive musculoskeletal injuries. DCO aims to mitigate the incidence of multiorgan failure and adult respiratory distress syndrome (ARDS) in patients who undergo early fixation after severe trauma. In these extreme conditions, EF may be favored as a means of initial stabilization of the DRF, decreasing the amount of injury related to swelling and edema, until the patient can be transported to an aseptic environment for debridement and definitive treatment [13]. Mathieu et al. illustrate the necessity of early stabilization with EF followed by subsequent conversion to ORIF [14]. The landscape of battlefield orthopedic care introduces distinctive challenges, characterized by high trauma severity, limited fixation resources, and challenging aseptic conditions confronting frontline surgical teams [14]. Consequently, adopting a staged treatment approach in line with DCO principles emerges as the most suitable strategy in this context. Ultimately, the authors of the referenced study deduced that a combination of minimally invasive EF, effective wound care, and timely transition to ORIF not only enhanced bone healing but also expedited recovery in combat-related limb injuries [14].

Short-term splinting for 48 hours is not indicated in cases of severe comminuted fractures, polytrauma patients, or those with significant soft tissue compromise. In such scenarios, the use of EF is preferable due to its ability to stabilize the fracture without compromising the injured area further. These principles can also find application in less extreme situations, such as instances where immediate IF instrumentation is unavailable at medical facilities. This can occur at small community hospitals, understaffed areas during nontraditional work hours, and other scenarios. Such conditions may lead to the utilization of either definitive EF for DRFs or a strategic progression from EF to IF, pending the patient’s transfer to a more equipped location. In such cases, it remains crucial to ascertain the optimal timing of ORIF and explore the viability of a later conversion from EF to IF [15]. Cross and Swiontkowski highlight the safety of transitioning from EF to IF as long as the conversion happens within a two-week timeframe and there is an absence of pin-site infections [16,17]. If conversion occurs after a pin site infection, it might necessitate additional time and antibiotic treatment following the removal of the EF before proceeding to IF [17]. This temporal consideration hinges significantly on the fracture healing process. The initial two weeks foster hematoma formation at the fracture site, followed by soft callus formation in subsequent weeks. To expedite healing and promote a faster return to baseline function, definitive alignment through IF should be initiated prior to the onset of fibrocartilage development [18]. It is important to note that pins located far beyond the surgical site, even if infected, are not a contraindication for definitive ORIF, as a pin-site infection is typically locally limited and manageable.

Another critical scenario arises when employing initial EF stabilization in physiologically unstable polytrauma patients, effectively postponing definitive treatment until achieving medical stability. EF can be utilized under local anesthesia with an average application time of just 20 minutes [19]. This quick approach mitigates the potential for fracture-induced compartment syndrome, curbing the impact of an extended period of internal fracture fixation, and reducing additional intraoperative blood loss. This strategy is especially significant in patients requiring multiple surgeries, as the overall risk of infection escalates. By implementing a delayed fixation for such fractures, the overarching prognosis for these patients can be optimized.

Soft Tissue Compromise

The management of fractures under compromised soft tissues requires a comprehensive approach that addresses both bone and soft tissue damage in the affected extremity. In the case of open fractures, proper debridement of the affected soft tissue and prompt administration of IV antibiotics are crucial to reduce complications and preserve viable tissue. EF has been shown to be invaluable for managing severely contaminated open DRFs and those with soft tissue damage [17]. To prevent further complications such as infection, osteomyelitis, and limb amputation, experts recommend early skeletal stabilization using EF, deferring definitive treatment until soft tissue swelling and injury are effectively managed [20].

It is important to note that IF after debridement has been demonstrated to be a safe and effective treatment for Gustilo Type I-II fractures [20]. Watson et al. describe IF management as yielding excellent results in complex fractures with minimal soft tissue compromise [21]. However, in complex Gustilo Type III & IV comminuted open fractures accompanied by severe soft tissue compromise, outcomes have been less favorable with higher complication rates, including infections, soft tissue breakdown, non-union, malunion, and secondary arthrosis [20]. These issues arise not only from the degree of wound contamination, but also from the interaction between immediate IF instrumentation facilitating extra swelling, trauma, and further compromise to the soft tissue. Even staged DCO techniques have shown to not be as optimal in treating these types of fractures, necessitating further corrective procedures [22].

In these severe open fractures, specifically Gustilo Type IIIA-C fractures, with extensive soft tissue compromise, EF has demonstrated the most favorable outcomes [17,20]. EF helps mitigate iatrogenic neurovascular injury and further compromise of the soft tissue [20]. By keeping the injury site free from instrumentation, EF’s minimally invasive approach allows for adequate bone reduction and improved wound access for further irrigation, debridement, and assessment.

Numerous studies in the orthopedic and trauma literature also underscore the favorable outcomes of EF in addressing additional instances of soft tissue compromise, including severe swelling and burns. Research by Serror et al. demonstrated that the adjustable nature of EF is particularly beneficial in accommodating the dynamic changes in soft tissue conditions associated with burns, while Bradshaw et al. highlight that IF cannot be a safe and effective option for those with these types of soft tissue compromise [23,24].

These collective findings affirm the positive impact of EF on soft tissue compromise, providing valuable insights for clinicians in optimizing treatment strategies for such challenging cases.

Comparing external fixation to internal fixation techniques

Anatomical Reduction, Radial Inclination, Volar Tilt, DASH Scores, and Grip Strength

The use of IF for the treatment of DRFs has expanded drastically with the ever-evolving landscape of innovative ORIF plates and screws. Multiple studies have evaluated the benefits of IF for the treatment of DRFs, demonstrating circumstances where the outcomes may seem to be superior to those seen by patients treated via EF. For example, Zamzuri et al. evaluated 26 patients to examine the outcomes of EF versus IF following DRFs and found greater anatomical reduction for patients treated via IF at both six months and 12 months postoperatively (p<0.05) [25]. Radiographic evidence of superior fixation has also been established, revealing improved anatomic radial inclination (p=0.04) and volar tilt (p=0.01) at 12 months postoperatively [26].

However, a systematic review of 10 randomized control trials (RCTs) by Xie et al. demonstrated that while IF provided better functional outcomes than EF, via Disabilities of the Arm, Shoulder, and Hand (DASH) scores, at three and six months postoperatively (p<0.001), the differences were resolved by 12 months [26]. Many additional studies have echoed similar findings of superior DASH scores for IF than EF at early follow-up time points, but all these differences seem to subside towards 12- and 24-month follow-up periods [6,7]. For patients where EF may be deemed appropriate, this modality has even been able to demonstrate in one study no difference in the patient-reported DASH score at any time point, including preoperatively (p=0.9), seven weeks postoperatively (p=0.5), three months postoperatively (p=0.6), and 12 months postoperatively (p=0.2) [27].

Providing specific subgroup analysis, Wei et al. revealed IF provided better DASH scores than EF when volar locking plates (VLP) were used (p=0.02) as opposed to when non-locking volar plates or non-volar plates were employed [28]. The use of VLP then demonstrated superior grip strength (p=0.01), supination (p<0.001), and extension (p<0.001) at three months postoperatively, yet again these differences resolved compared to EF at subsequent follow-up time points [26,28,29]. Furthermore, Huang et al. evaluated 69 patients over 80 years old with DRFs treated via VLP (n=21) or EF (n=41). Radiographically they found VLP provided a reduction in ulnar variance (p=0.01) with functional outcomes displaying greater supination at final follow-up (p=0.002) [30]. However, EF was shown to have promising outcomes with regard to radial length restoration and alignment. Prommersberger et al. highlighted that compared to VLP, EF of DRFs minimizes the risk of both malunions and non-union when there is a shortening of the radius in relation to the ulna [31].

Postoperative physical function benefits have been extensively evaluated through many more systematic reviews and meta-analyses. Wei et al. found patients treated by EF demonstrated increased grip strength (standardized mean difference or SMD, -0.28; 95% CI, -0.57 to 0.00; P = 0.05; I2 = 68%) with a further subgroup analysis revealing EF was more favorable than VLP (SMD, -0.33; 95% CI, -0.58 to -0.08; P = 0.01; I2 = 29%) [28]. Significantly better grip strength following EF, with improved mean strength of 10.1 pounds, was also seen in a comparison study of 88 patients who underwent indirect fixation and 91 who underwent IF (95% CI, 1.9 to 18.3, p = 0.05) [32]. It should be noted again that many differences between the different fixations resolve after six months postoperatively [32].

Additional Considerations 

Although many of these previously mentioned studies allude to promising EF results, caution should still be used when employing the use of EF. Margaliot et al. evaluated 48 studies through a meta-analysis and found higher rates of neuritis, hardware failure, and infection for patients treated with EF, while also concluding there was no appreciable difference in grip strength between IF and EF cohorts [33]. Overall, when considering the outcomes of IF versus EF, we can observe better EF grip strength and more rapid improvements from the IF range of motion (ROM); yet, many of these improvements dissipate with time, revealing equivocal outcomes [25,26,28,29].

Once thorough debridement has occurred, EF can offer stabilization while avoiding additional internal hardware as the patient is started on high-dose antibiotics. As DRF non-unions and infections are rare, limited research exists to identify the best method for treating these complex cases. Additional study in this area is warranted.

When it comes to cost, IF and EF meet similar measures. Rajan et al. developed a Markov model that projected costs in patients undergoing fixation and found that procedural costs were $7,638 for closed reduction and percutaneous pinning (CRPP), $10,170 for ORIF, and $9,886 for EF [9]. From the healthcare payer perspective, total costs were $8,735 (CRPP), $11,125 (ORIF), and $11,759 (EF) and from the societal perspective, total costs were $19,435 (CRPP), $19,214 (ORIF), and $22,295 (EF) [9]. The cost was then twofold if a fracture utilized temporary EF and needed an operative return for IF [9]. Yet, Yoon et al. concluded from their study that the total cost expended was $6,837 for casting, $11,329 for CRPP, $16,012 for EF, and $16,354 for VLP [34]. These similarities in outcomes and cost between EF and IF suggest that healthcare providers can consider a more flexible approach in their surgical decision-making.

Comparing external fixation to bridge plating

The original introduction of the dorsal bridge plate technique in 1998 aimed to address fractures of the distal radius that involved significant damage to the joint surface or extended to the meta-diaphyseal region [10]. This technique involved a closed or limited open reduction of the fracture, followed by placing a long straight dorsal bridge plate beneath the wrist's second or fourth dorsal compartments. The plate is fixed to the proximal dorsal radial shaft and the distal index or middle metacarpal shaft using multiple screws. In essence, this technique functions as an "internal external fixator," spanning both the fracture and the wrist joint. However, it offers the additional advantage of IF, which theoretically reduces the risk of complications associated with external pins and hardware. Over time, various authors have refined the technique, expanding its application to include managing osteoporotic fractures of the distal radius, fractures with extensive joint damage or meta diaphyseal comminution, and cases involving radiocarpal instability.

There are various advantages to using a bridge plate to treat distal radius fractures. It is an improved fixation technique that allows comminuted articular fragments to reduce under ligamentotaxis and provide a buttress for the dorsal cortex of the distal radius [5]. The long dorsal plate can bridge past metaphyseal comminution, which cannot be so easily addressed with standard volar plating. The bridge plate can also be left in place for an extended period without the risk of pin-track infections and major hardware discomfort associated with EF use [5]. Finally, one author believes the dorsal distraction plate is a less technically demanding operation to perform than volar plating for complex articular fractures [5].

Compared to EF, dorsal bridge plating has demonstrated lower rates of infection and complex regional pain syndrome, but higher rates of hardware failure [35]. Bridge plating also demonstrated higher rates of excellent measures under the Gartland and Werley outcome score. Bridge plating also allows for immediate weight bearing if all screw holes are filled, which may be an advantageous consideration when deciding on a fixation technique in a polytrauma setting [10]. In the setting of complex DRFs, dorsal bridge plating was found to have stronger grip strength, lower DASH scores, and lower Visual Analog Scale at three-month follow-up compared to EF [36]. But, again, it is important to note that at the 12-month follow-up, there was no significant difference in terms of radiographic parameters or wrist range of motion. Although some advantages exist, like the ability to allow for immediate weight bearing, dorsal bridge plating has still demonstrated higher hardware failure rates when compared to EF [36].

Unique scenarios and demographics

Elderly Patients

There are additional DRF patterns and patient demographics for which providers might want to consider EF. Elderly patients often present with significant comorbidities, particularly cardiac issues, which can give orthopedic surgeons pause when considering surgery. Traditionally, their comorbid conditions and osteoporotic bone density lead providers to lean toward non-operative treatments, especially for extra-articular DRFs. However, several studies, like Orbay et al. have observed that operative fixation for intra-articular DRFs in this demographic can yield successful outcomes over time [37].

Arora et al. conducted a comparative study evaluating the clinical and radiological outcomes of two treatment approaches for unstable DRFs in patients over 70 years old [38]. Their research indicated that patients treated with IF using a VLP exhibited significantly better radiographic results, including dorsal tilt, radial inclination, and radial shortening, when compared to those managed with cast immobilization [38]. Expanding on this comparison, Ma et al. examined VLP versus EF in patients over 65 years old [12]. Their findings indicated that IF offered advantages in the early rehabilitation phase, but after one year, both approaches yielded similar outcomes. It’s worth noting that the plating group experienced significantly higher rates of wound infection and tendonitis, necessitating more additional surgeries [12].

Aita et al. observed that both bridging and non-bridging EF proved to be safe and dependable for treating unstable DRFs in elderly patients with polytrauma. Importantly, the grip strength outcomes in both groups correlated with the restoration of independent daily living capabilities in the elderly [39]. Moreover, Cheng et al. emphasized the reliability of the hybrid non-bridging EF technique for managing unstable extra-articular and simple intra-articular DRFs in patients aged over 50 years old [40]. These studies collectively contribute valuable insights into the management of DRFs in the elderly, highlighting the nuances between treatment modalities and their long-term complications.

Non-unions and Malunions

Another intriguing patient cohort to consider for EF includes individuals with non-unions or malunions necessitating corrective osteotomies or debridement for infection. Shin and Jones illuminate the effectiveness of the Agee-Wristjack EF in offering provisional stabilization at the osteotomy site for these distal radius bone defects [41]. This stable distraction greatly facilitates the precise alignment of the distal fragment and permits the harvesting and meticulous shaping of iliac crest bone grafts to precisely fit the osteotomy site. Furthermore, opting to place pins into the index metacarpal, rather than the distal fragment, streamlines the application of a plate, minimizing interference from pins. The Agee-Wristjack EF can be kept in place for up to six weeks to help stabilize the IF [41].

Similarly, Ponnanna et al. advocate for the successful utilization of EF in conjunction with the modified Sauve-Kapandji techniques following infected IF, enabling the proper debridement of a malunion [42]. In addition, the wrist-spanning external fixator can be kept in place for six weeks to help maintain radial height [42]. These studies collectively substantiate the notion that EF can complement IF when managing these intricate and complicated cases.

Bilateral Distal Radius Fractures

Another interesting patient cohort where providers could consider EF is patients with bilateral distal radius fractures. While rare, bilateral distal radius fractures present unique challenges to the patient. As described by Dağtaş and Ünal hand function in the first two months post-surgery was better in the EF group versus the ORIF group [43]. At day 15 and months 1 and 2, the Q-DASH score was lower for the EF group but became similar between the groups by year 1. The Mayo wrist score was higher in the EF group at all time periods measured during the first year (15 days, 1 and 2 months, and 1 year) [43].

Similarly to the other studies discussed above, Dağtaş and Ünal reported a longer operation time for the ORIF group (p<0.001) [43]. While the EF group had complications in 31.8% of cases versus 25.0% for the ORIF group, no statistical significance in Müller’s Arbeitsgemeinschaft für Osteosynthesefragen (AO) classification and complication development was seen between the groups [43]. These findings highlight EF should be considered as a possible treatment of bilateral DRF, especially when more rapid short-term results are needed.

Different types of external fixation

While patient and fracture characteristics undoubtedly play a crucial role in determining the most appropriate treatment, a similar level of consideration is warranted when selecting the type of EF technique. There currently exist two primary categories: non-bridging and bridging EFs. The comparison between the two in the management of DRFs reveals distinct advantages and considerations. Unlike conventional bridging EF, where the distal pins span the wrist joint, non-bridging fixators are placed solely into the distal fracture fragment, avoiding joint involvement.

In a randomized prospective study conducted by McQueen in 1998, these two approaches were meticulously evaluated [44]. Non-bridging fixation emerges as the preferred choice when at least 1 cm of the volar cortex is present in the distal fragment or articular displacement [44]. The non-bridging group demonstrated superior maintenance of normal volar tilt, carpal alignment, grip strength, and flexion [44]. Notably, one year after injury, the bridging fixator group exhibited a mean dorsal tilt of 12.2 degrees, contrasting with the 5.6 degrees seen in the non-bridging group [44]. The authors underlined a key advantage of non-bridging fixation, as it avoids wrist joint distraction, allowing for enhanced range of motion during both fixator placement and post-fixator removal.

Similarly, in a 2020 comparative analysis by Liu and Bai, non-bridging EF continued to emerge as the superior option [45]. From the patient’s standpoint, non-bridging EF recipients were more likely to report less pain, fewer disabilities, fewer limitations, and improved wrist joint function at the 12-week mark. Furthermore, measurements of radial inclination, length, and palmar tilt at the 12-week assessment favored non-bridging EF over bridging EF [45]. Additionally, non-bridging EF has also demonstrated a lower incidence of complex regional pain syndrome in contrast to bridging EF [46]. Notably, Atroshi et al., in a smaller randomized study, found that non-bridging was particularly effective at preserving radial length in cases of displaced DRFs [47]. These studies underscore the pivotal role of non-bridging EF in enabling orthopedic surgeons to conserve a greater portion of a patient’s wrist structure and function when compared to bridging EF for definitive treatment.

Future research

Numerous unresolved questions persist regarding the optimal application of EF in the management of DRFs. Extensive research endeavors are essential to explore the comparative effectiveness of temporary splinting versus temporary EF. Remarkably, there exist accounts of instances where elderly patients, beset by comorbidities, have notable benefits from a temporary EF approach until definitive VLP treatment could be initiated, underscoring the need for further investigation in this context. Moreover, further research should delve into the specific fracture patterns and demographics of both patients and surgeons that are most amenable to definitive DRF fixation with only an external fixator. This can provide invaluable insight into tailoring treatment strategies to individualized needs. Furthermore, additional studies are warranted to establish comprehensive guidelines regarding intra-articular fractures that should categorically avoid EF as a treatment option. Such research initiatives promise to refine and optimize the utilization of EF in the comprehensive management of DRFs, ultimately enhancing patient outcomes and reducing treatment variability.

## Conclusions

Current literature emphasizes that most orthopedic surgeons employ internal fixation for numerous distal radius fractures, with a particular focus on volar locking plates and bridge plating. However, there exist several clinical scenarios that may benefit from being managed with external fixation (EF). These include the setting of damage control orthopedics when managing patients with severe soft tissue swelling, or markedly comminuted and unstable fractures in the elderly, which may be appropriately managed with EF. Future research should aim to produce high-level studies that compare outcomes amongst the different modes of internal fixation and EF in a variety of clinical settings to best understand the most appropriate use of these techniques.
